# Prognostic value of the hemoglobin, albumin, lymphocyte, and platelet score for short-term mortality in dogs with meningoencephalitis of unknown etiology

**DOI:** 10.3389/fvets.2026.1843876

**Published:** 2026-06-05

**Authors:** Sejung Ahn, Yeon Chae, Yoonhoi Koo, Hakhyun Kim, Byeong-Teck Kang, Taesik Yun

**Affiliations:** 1 Laboratory of Veterinary Internal Medicine, College of Veterinary Medicine, Chungbuk National University, Cheongju, Chungbuk, Republic of Korea; 2 College of Veterinary Medicine, Kyungpook National University, Daegu, Republic of Korea

**Keywords:** canine, HALP score, MUE, MUO, prognosis, short-term mortality

## Abstract

Meningoencephalitis of unknown etiology (MUE) is a common inflammatory central nervous system disease in dogs and is associated with a poor prognosis. The first 3 months post-diagnosis represent a critical prognostic window, as most mortalities occur within this period. The hemoglobin, albumin, lymphocyte, and platelet (HALP) score has emerged as a novel prognostic tool for predicting short-term mortality in various human diseases. However, its utility has not yet been evaluated in dogs with MUE. This retrospective study aimed to evaluate the predictive value of the HALP score for three-month and one-year mortality in 36 dogs diagnosed with MUE. The HALP score was significantly higher in survivors than in non-survivors at both the three-month and one-year time points. Receiver operating characteristic curve analysis demonstrated good predictive accuracy, with an area under the curve of 0.79 for three-month and 0.80 for one-year mortality. An optimal cut-off of 12.25 yielded 50.0% sensitivity and 95.0% specificity for predicting three-month mortality. For one-year mortality, a cut-off of 34.33 yielded 95.2% sensitivity and 66.7% specificity. Dogs with a HALP score ≤ 12.25 had a significantly shorter median survival time (31 days) than those with a score > 12.25 (283 days). Similarly, a score ≤ 34.33 was associated with a shorter median survival time (67.5 days) compared to those with a score > 34.33 (971 days). These findings suggest that the HALP score is a valuable, non-invasive indicator for risk stratification and predicting short-term mortality in dogs with MUE.

## 1 Introduction

Meningoencephalitis of unknown etiology (MUE) is one of the most common non-infectious, inflammatory conditions of the central nervous system (CNS) in dogs, characterized by a broad spectrum of clinical signs and manifestations (1, 2). Although its exact pathophysiology remains unclear, an autoimmune etiology is suspected (3). The prognosis for MUE is often poor, even with aggressive immunosuppressive therapy (4).

The first 3 months post-diagnosis represent a critical prognostic window, as most mortalities occur within this period (5–7). Dogs that survive beyond this initial phase have a significantly higher likelihood of reaching 1 year, and survival to 1 year is often associated with a favorable long-term outcome (5, 8).

Several prognostic factors for MUE have been investigated, including cerebrospinal fluid (CSF) analysis, imaging features, and seizure status. However, the findings are often conflicting. For instance, while lower CSF total nucleated cell counts have been linked to improved survival in some studies (9), others report no prognostic value for CSF abnormalities (8, 10). Similarly, imaging features such as mass effect and foramen magnum herniation are associated with increased mortality (6), yet the significance of other findings like midline shift remains inconsistent (9, 11). Seizure at presentation is a more consistent predictor of shorter survival times (10, 12, 13). Despite these efforts, the available prognostic information for MUE remains inconsistent and difficult to apply in clinical practice. Furthermore, there is a notable lack of research into hematological parameters as prognostic indicators in canine MUE. Therefore, highlighting the prognostic value of these parameters is crucial for addressing this knowledge gap and expanding clinical diagnostic options. Consequently, a clear need exists to identify objective, easily accessible biomarkers that can reliably predict outcomes to improve the management of dogs with MUE.

The hemoglobin, albumin, lymphocyte, and platelet (HALP) score has recently emerged as a promising prognostic marker in human medicine, predicting short-term mortality in various conditions such as malignant tumors, pancreatitis, acute heart failure, and acute ischemic stroke (14–20). As a simple and non-invasive tool, the HALP score reflects a patient's overall nutritional status, immune function, and systemic inflammatory response (21, 22).

To our knowledge, the prognostic value of the HALP score has not yet been explored in veterinary medicine, particularly for canine MUE. This study, therefore, was designed to investigate the utility of the HALP score for predicting three-month and one-year mortality in dogs with MUE. We hypothesized that a higher HALP score would be associated with improved survival outcomes.

## 2 Methods

### 2.1 Animals

**Figure 1:**
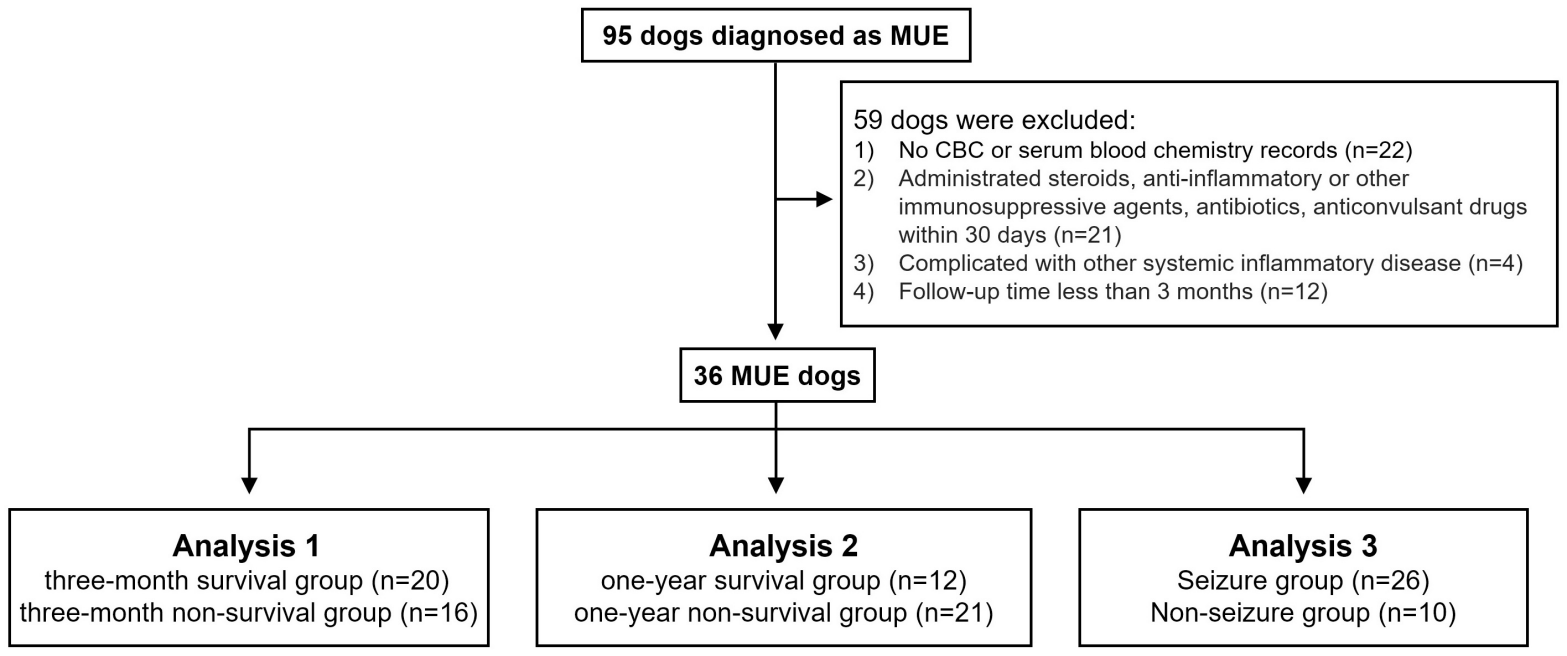
Flow diagram illustrating the inclusion and exclusion of dogs with MUE. CBC, complete blood count; MUE, meningoencephalitis of unknown etiology.

This retrospective study involved a review of medical records from dogs diagnosed with MUE at the Veterinary Teaching Hospital of Chungbuk National University between January 2014 and February 2024. The case selection process is summarized in Figure 1. A total of 36 dogs were included. A definitive diagnosis of MUE is made through histopathology, whereas a presumptive diagnosis of MUE without histopathology was made based on the criteria established in a previous study (13), which required: (i) age over 6 months; (ii) focal or multifocal neurologic signs; (iii) hyperintense lesions on T2-weighted and fluid-attenuated inversion recovery (FLAIR) images; (iv) mononuclear pleocytosis in CSF; and (v) exclusion of infectious etiologies via CSF cytology and polymerase chain reaction tests (*Cryptococcus* spp., *Bartonella* spp., *Histoplasma* spp., *Blastomyces* spp., *Anaplasma* spp., *Ehrlichia* spp., *Neospora* spp., *Borrelia burgdorferi, Toxoplasma gondii*, and Canine distemper virus).

The exclusion criteria were as follows: (i) incomplete medical records; (ii) administration of anti-inflammatory, immunosuppressive, anticonvulsant drugs, or antibiotics within the 30 days prior to diagnosis; and (iii) presence of concurrent diseases known to affect white blood cell counts or albumin levels, such as hyperadrenocorticism, lymphoma, or chronic kidney disease.

### 2.2 HALP score calculation

For each dog, blood samples were collected at the initial visit for a complete blood count (CBC) and a serum chemistry profile. Whole blood was collected in ethylenediaminetetraacetic acid tubes for the CBC, and blood for the chemistry profile was collected in serum separator tubes. All samples were processed promptly; serum was separated via centrifugation within 30 min of collection, and both CBC and chemistry analyses were performed immediately thereafter. The CBC was conducted using an automated hematology analyzer (ProCyte Dx, IDEXX Laboratories, Inc., Westbrook, ME, USA) to determine hemoglobin concentration, lymphocyte count, and platelet count. Serum albumin concentration was measured with a biochemical analyzer (Hitachi 7020, Hitachi High-Technologies Co., Tokyo, Japan). The HALP score was then calculated using the following formula:


$$\begin{array}{l} \text{HALP Score = }(\text{Hemoglobin }[\text{g/L}]\;\times \text{ Albumin }[\text{g/L}]\\ \;\times \text{ Lymphocyte count }[\text{/L}])\text{/Platelet count}\;(\text{/L}). \end{array}$$

The components of the HALP score were selected to provide a comprehensive assessment of the patient's systemic status; hemoglobin and albumin serve as reliable indicators of nutritional status and systemic tissue oxygenation, while lymphocyte and platelet counts reflect the host's immune surveillance and systemic inflammatory response, respectively.

### 2.3 Brain lesion volume estimation

All dogs underwent a brain magnetic resonance imaging scan using either a 0.3-T (Airis II, Hitachi, Tokyo, Japan) or a 1.5-T (Signa Creator, GE Healthcare, Milwaukee, WI, USA) scanner. The imaging protocol included transverse, sagittal, and dorsal planes with T1-weighted (pre- and post-contrast), T2-weighted, and FLAIR sequences.

MUE lesions were manually delineated on transverse FLAIR images using a commercial image viewer (OsiriX MD v10.0, Pixmeo Sarl, Geneva, Switzerland). The total lesion volume was then calculated by summing the area of the lesion on each consecutive slice and multiplying by the slice thickness.

### 2.4 Outcome assessment

The primary outcomes of this study were three-month and one-year mortality. These specific endpoints were selected because the first 3 months post-diagnosis represent a critical prognostic window where most mortalities occur in dogs with MUE (5–7), whereas one-year survival is strongly associated with favorable long-term outcomes (5, 8). These endpoints were used to evaluate the clinical significance and prognostic value of the HALP score in dogs with MUE. Among the 36 dogs, survival status and cause of death for 34 dogs were confirmed through clinical examination during follow-up visits at our institution. For the remaining two dogs, the survival data were obtained via telephone interviews, focusing on the date of death and owner-reported clinical status.

### 2.5 Statistical analyses

All statistical analyses were conducted using Prism 10.0 (GraphPad Software Inc., La Jolla, CA, USA). The normality of continuous variables was assessed with the Shapiro-Wilk test. Normally distributed data are presented as mean ± standard deviation and were compared using an unpaired *t*-test. Non-normally distributed data are presented as median (interquartile range) and were compared using the Mann-Whitney U test. Categorical variables, presented as frequencies (n) and percentages (%), were compared using the Pearson's chi-square test. The predictive utility of the HALP score for three-month and one-year mortality was evaluated using receiver operating characteristic (ROC) curve analysis. The area under the curve (AUC) was calculated to assess diagnostic accuracy, classified as: sufficient (0.6–0.7), good (0.7–0.8), very good (0.8–0.9), or excellent (0.9–1.0). The optimal cut-off value was determined by the Youden J index, for which sensitivity and specificity were calculated. Survival analysis was performed using the Kaplan-Meier method, with survival curves compared using the log-rank test. A *P*-value < 0.05 was considered statistically significant for all analyses.

## 3 Results

### 3.1 Study population

The study included 36 dogs diagnosed with MUE. Detailed demographic characteristics are provided in Table 1. The cohort was predominantly composed of Maltese dogs (*n* = 20, 55.6%), with other breeds including Chihuahua (*n* = 4, 11.1%), Yorkshire Terrier (*n* = 3, 8.3%), Miniature or Toy Poodle (*n* = 3, 8.3%), mixed-breed (*n* = 3, 8.3%), Pomeranian (*n* = 2, 5.6%), and Miniature Pinscher (*n* = 1, 2.8%). The mean age of the dogs at diagnosis was 5.97 ± 3.44 years. The group included 17 males (6 intact, 11 castrated) and 19 females (9 intact, 10 spayed). Seizures were a presenting clinical sign in 26 of the 36 dogs (72.2%). The overall mortality rates at 3 months and 1 year were 44.4% and 63.6%, respectively. All 36 dogs diagnosed with MUE received immunosuppressive therapy tailored to their clinical response. Prednisolone was prescribed as the core component of the treatment protocol for all patients. Regarding the use of secondary immunosuppressive agents, four dogs (11.1%) received prednisolone monotherapy. The majority of the cohort (*n* = 21, 58.3%) was treated with a combination of prednisolone and mycophenolate mofetil. Other secondary agents used included cyclosporine (*n* = 5, 13.9%) and cytosine arabinoside (*n* = 2, 5.6%). In four cases (11.1%), the secondary agent was switched during the follow-up period due to clinical considerations or adverse effects.

**Table 1:** Demographic characteristics of dogs with MUE.

Variables	MUE dogs (*n* = 36)
Age (years)	5.972 ± 3.435
Sex	
Intact male	6 (16.7%)
Castrated male	11 (30.5%)
Intact female	9 (25%)
Spayed female	10 (27.8%)
Seizure	
Present	26 (72.2%)
Absent	10 (27.7%)

Age is presented as mean ± standard deviation. Sex and seizure status are presented as the number of dogs (%). MUE, meningoencephalitis of unknown etiology.

### 3.2 Comparison of HALP score with mortality and seizure status

**Figure 2:**
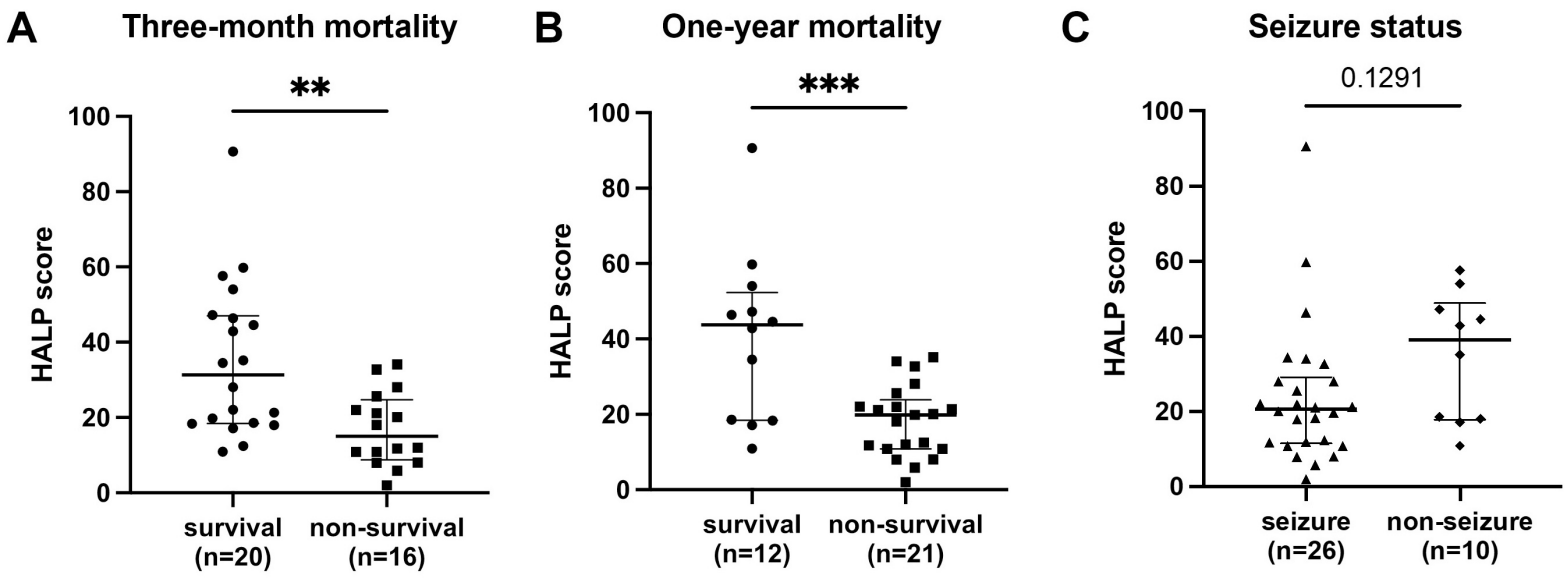
Comparison of HALP scores by mortality and seizure status. The HALP score was significantly higher in dogs that survived compared to those that did not at both **(A)** 3 months and **(B)** 1 year. **(C)** No significant difference was found between dogs with and without seizures. Each point represents an individual dog, and horizontal lines indicate the median and interquartile range for each group. **P < 0.01, ***P < 0.001. HALP, hemoglobin, albumin, lymphocyte, and platelet; MUE, meningoencephalitis of unknown etiology.

Comparisons of demographic and clinical data based on three-month mortality, one-year mortality, and seizure status are detailed in Tables 2–4, respectively. The HALP score was significantly higher in dogs that survived compared to those that did not, at both the three-month (*P* = 0.0025; Figure 2A) and one-year (*P* = 0.0004; Figure 2B) time points. In addition to the HALP score, survivors also had significantly higher hemoglobin (three-month: *P* = 0.0019; one-year: *P* = 0.0053) and serum albumin concentrations (three-month: *P* = 0.0325; one-year: *P* = 0.0010). The presence of seizures was also significantly associated with mortality at both time points (*P* = 0.0130 and *P* = 0.0046, respectively). Conversely, when comparing dogs with and without seizures, no significant differences were observed for any variables, including the HALP score (*P* = 0.1291; Figure 2C).

**Table 2:** Comparison of demographic and clinical data according to three-month mortality.

Variables	Survival (*n* = 20)	Non-survival (*n* = 16)	*P*-value
Sex (number)			0.3899
Intact male	3	3	
Castrated male	4	7	
Intact female	6	3	
Spayed female	7	3	
Age (years)	5.75 ± 3.754	6.25 ± 3.088	0.6707
Seizure (number)			0.0130^*^
Present	11	15	
Absent	9	1	
Hemoglobin (g/L)	168.5 ± 27.91	138.3 ± 25.16	0.0019^*^
Albumin (g/L)	34.85 ± 5.833	30.81 ± 4.792	0.0325^*^
Lymphocyte (10^9^/L)	1.870 (1.020–2.608)	1.465 (0.70–2.575)	0.2291
Platelet (10^9^/L)	286.5 (247.5–485.5)	390.5 (279.8–503.8)	0.5312
HALP score	31.30 (18.44–47.01)	15.04 (8.718–24.71)	0.0025^*^
Lesion volume (cm^3^)	2.900 (1.330–7.830)	2.086 (0.3980–3.909)	0.2114

Normally distributed continuous data are presented as mean ± standard deviation and were compared between groups using an unpaired *t*-test. Non-normally distributed continuous data are presented as median (interquartile range) and were compared using the Mann-Whitney U test. Categorical data are presented as number of dogs and were compared using the Pearson's chi-square test. ^*^Indicates a statistically significant difference between groups. HALP, hemoglobin, albumin, lymphocyte, and platelet.

**Table 3:** Comparison of demographic and clinical data according to one-year mortality.

Variables	Survival (*n* = 12)	Non-survival (*n* = 21)	*P*-value
Sex (number)			0.7858
Intact male	2	4	
Castrated male	3	8	
Intact female	3	5	
Spayed female	4	4	
Age (years)	4.833 ± 3.380	6.048 ± 2.941	0.2880
Seizure (number)			0.0046^*^
Present	5	19	
Absent	7	2	
Hemoglobin (g/L)	173.6 ± 31.36	143 ± 26.16	0.0053^*^
Albumin (g/L)	37.33 ± 4.376	30.71 ± 5.386	0.0010^*^
Lymphocyte (10^9^/L)	2.157 ± 1.397	1.662 ± 0.8802	0.2190
Platelet (10^9^/L)	286.5 (238.8–454.5)	384.0 (274.0–523.5)	0.3904
HALP score	40.43 ± 22.56	18.19 ± 9.491	0.0004^*^
Lesion volume (cm^3^)	2.1250 (0.8838–7.4030)	2.1320 (0.4122–4.2020)	0.7261

Normally distributed continuous data are presented as mean ± standard deviation and were compared between groups using an unpaired *t*-test. Non-normally distributed continuous data are presented as median (interquartile range) and were compared using the Mann-Whitney U test. Categorical data are presented as number of dogs and were compared using the Pearson's chi-square test. ^*^Indicates a statistically significant difference between groups. HALP, hemoglobin, albumin, lymphocyte, and platelet.

**Table 4:** Comparison of demographic and clinical data between seizure and non-seizure groups.

Variables	Seizure (*n* = 26)	Non-seizure (*n* = 10)	*P*-value
Sex (number)			0.6868
Intact male	4	2	
Castrated male	9	2	
Intact female	7	2	
Spayed female	6	4	
Age (years)	6 (3–8)	6 (3.5–8.5)	0.4389
Hemoglobin (g/L)	151.5 ± 29.70	164.4 ± 31.92	0.2593
Albumin (g/L)	33.00 ± 6.020	33.20 ± 5.051	0.9264
Lymphocyte (10^9^/L)	1.711 ± 0.9822	2.174 ± 1.289	0.2540
Platelet (10^9^/L)	353.0 (265.0–485.0)	286.5 (225.5–526.3)	0.5599
HALP score	20.63 (11.54–29.24)	39.02 (17.85–48.92)	0.1291
Lesion volume (cm^3^)	2.9470 (1.2070–8.0450)	1.4320 (0.3803–3.5780)	0.1886

Normally distributed continuous data are presented as mean ± standard deviation and were compared between groups using an unpaired *t*-test. Non-normally distributed continuous data are presented as median (interquartile range) and were compared using the Mann-Whitney U test. Categorical data are presented as number of dogs and were compared using the Pearson's chi-square test. HALP, hemoglobin, albumin, lymphocyte, and platelet.

### 3.3 Predictive performance of the HALP score for short-term mortality

**Figure 3:**
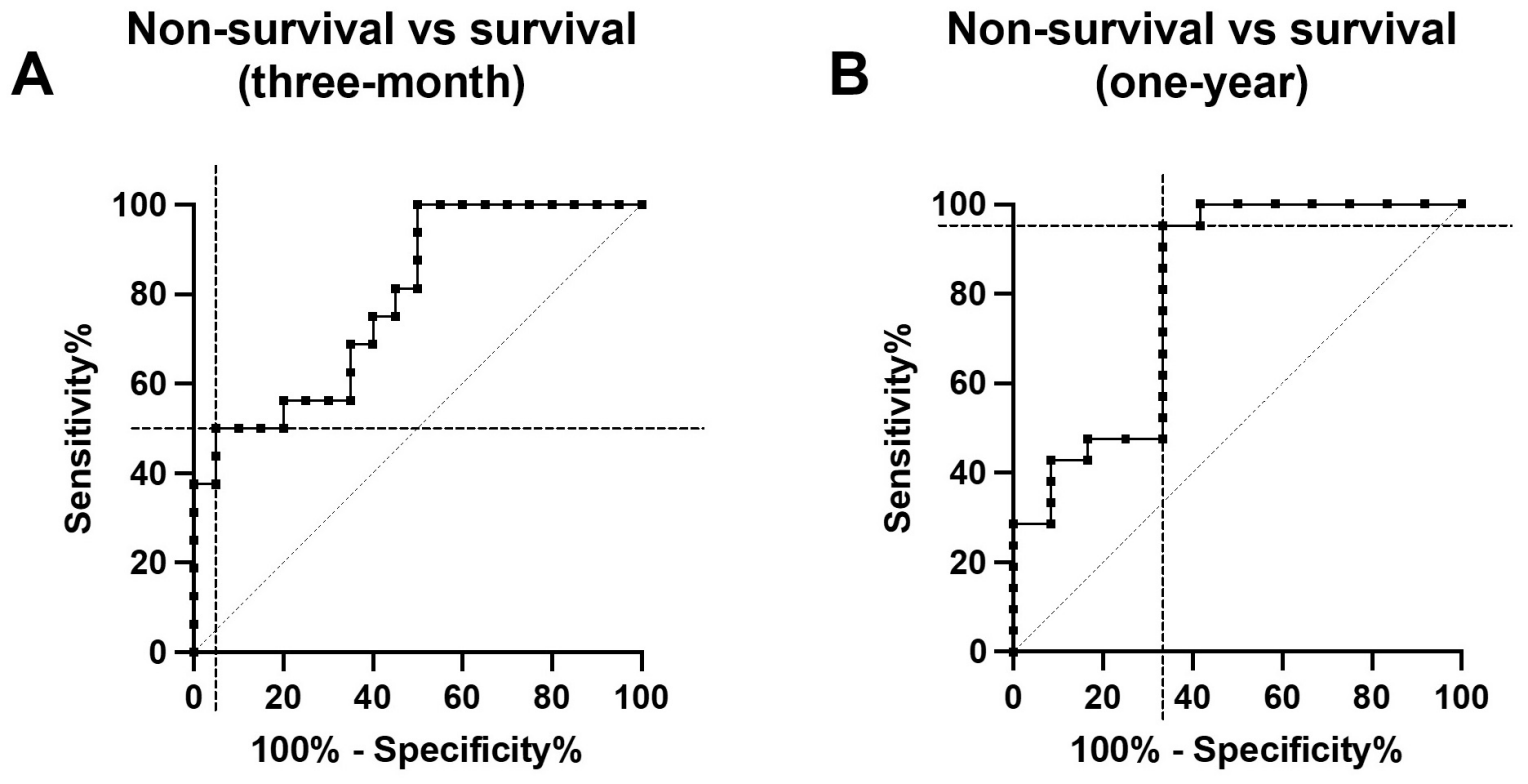
ROC curves for the HALP score in predicting mortality. The HALP score demonstrated good predictive accuracy for **(A)** three-month mortality (AUC = 0.79; 95% CI, 0.65–0.95) and **(B)** one-year mortality (AUC = 0.80; 95% CI, 0.63–0.97). The optimal cut-off for 3 month mortality was 12.25 (sensitivity: 50.00%, specificity: 95.00%). For 1 year mortality, the optimal cut-off was 34.33 (sensitivity: 95.24%, specificity: 66.67%). The diagonal line represents the line of no-discrimination (AUC = 0.5). AUC, area under the curve; CI, confidence interval; HALP, hemoglobin, albumin, lymphocyte, and platelet; MUE, meningoencephalitis of unknown etiology; ROC, receiver operating characteristic.

ROC curve analysis was performed to assess the predictive performance of the HALP score for three-month and one-year mortality (Figure 3). The HALP score demonstrated good predictive accuracy for both endpoints. For three-month mortality, the AUC was 0.79 (95% CI, 0.64–0.94). The optimal cut-off value was 12.25, which yielded a sensitivity of 50.00% and a specificity of 95.00%. For one-year mortality, the AUC was 0.80 (95% CI, 0.63–0.97). At the optimal cut-off of 34.33, the sensitivity was 95.24% and the specificity was 66.67%.

### 3.4 Survival analysis

**Figure 4:**
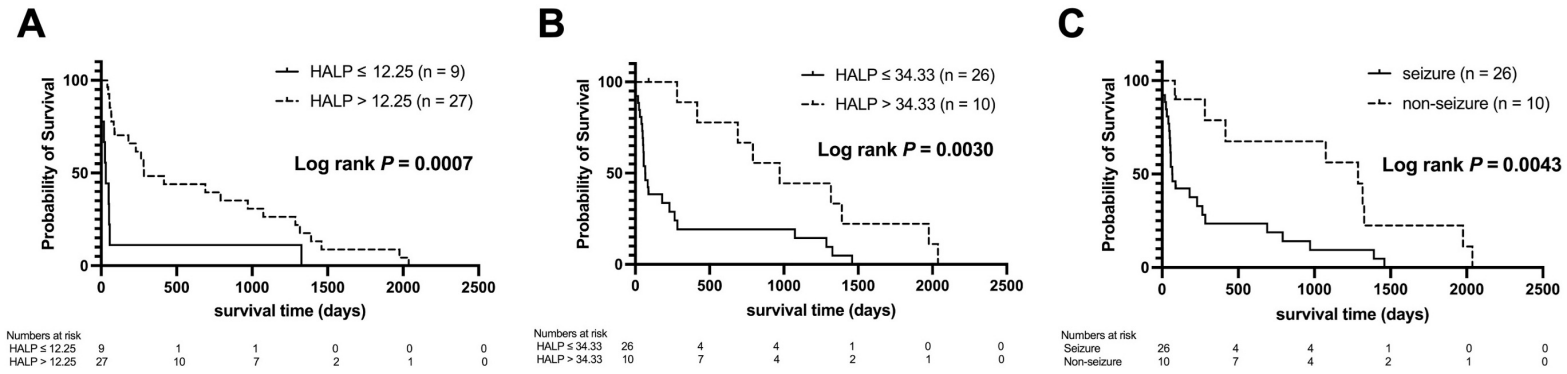
Kaplan-Meier survival analysis for dogs with MUE. Overall survival was significantly shorter for dogs with **(A)** a HALP score ≤ 12.25 (log-rank *P* = 0.0007), **(B)** a HALP score ≤ 34.33 (log-rank *P* = 0.0030), and **(C)** the presence of seizures (log-rank *P* = 0.0043). The number of dogs at risk at each time point is shown below the x-axis. HALP, hemoglobin, albumin, lymphocyte, and platelet; MUE, meningoencephalitis of unknown etiology.

Kaplan-Meier survival analysis demonstrated that a lower HALP score was significantly associated with decreased survival time. When stratified by the three-month mortality cut-off (12.25), dogs with a HALP score ≤ 12.25 had a significantly shorter median survival time of 31 days compared to 283 days for dogs with a score > 12.25 (log-rank *P* = 0.0007; Figure 4A). A similar significant difference was observed using the one-year mortality cut-off (34.33). The median survival time for dogs with a HALP score ≤ 34.33 was 67.5 days, vs. 971 days for those with a score > 34.33 (log-rank *P* = 0.0030; Figure 4B). Additionally, the presence of seizures was a significant negative prognostic factor, with the non-seizure group showing a significantly higher survival probability than the seizure group (log-rank *P* = 0.0043; Figure 4C).

## 4 Discussion

This study is the first to evaluate the HALP score as a prognostic indicator for short-term mortality in dogs diagnosed with MUE. Our findings clearly demonstrate that a lower HALP score is significantly associated with poorer survival outcomes. Specifically, the HALP score was significantly higher in dogs that survived compared to those that did not at both three-month and one-year follow-ups. This prognostic utility was further confirmed by survival analysis, where optimal HALP score cut-off values effectively stratified dogs into distinct survival probability groups. Therefore, the HALP score represents a valuable, accessible, and non-invasive biomarker for risk stratification in dogs with MUE.

In human medicine, the HALP score is an established prognostic tool across a range of diseases, including malignant tumors, pancreatitis, and stroke (14–20, 23). Its clinical utility is highlighted by specific examples. For instance, in patients with acute pancreatitis, the HALP score effectively predicts short-term mortality, demonstrating 82.8% sensitivity and 86.8% specificity (20). Likewise, in acute ischemic stroke, it predicts poor 90-day outcomes with 53.3% sensitivity and 75.9% specificity (18). These studies suggest the HALP score as a versatile prognostic marker in human medicine.

The current study of the HALP score in dogs with MUE is grounded in previous research that has identified MUE as a naturally occurring canine model for immune-mediated CNS diseases, including multiple sclerosis in humans (24, 25). In human patients with multiple sclerosis, free hemoglobin released due to erythrocyte fragility can damage the blood-brain barrier and exacerbate inflammation by triggering oxidative stress and activating the innate immune response (26). Following the breakdown of the blood-brain barrier, albumin is expected to access CNS tissue, resulting in a reduction in serum albumin concentration (27). Its leakage into CNS tissue may contribute to brain atrophy and the progression of disability (27). Anemia and decreased albumin concentration may occur through a similar mechanism in dogs with MUE. Moreover, prolonged severe inflammation may exacerbate reductions in both hemoglobin and albumin concentrations (28, 29). Additionally, MUE is recognized as an autoimmune disease characterized by autoantibodies targeting astrocytic antigens, leading to neuroinflammation and the accumulation of perivascular mononuclear cells marked by lymphocytic infiltration (30, 31). MUE is also characterized by the formation of perivascular cuffs composed of lymphocytes, plasma cells, and histiocytic cells, which are believed to contribute to reduced peripheral lymphocyte counts (31). Chronic lesions may infiltrate and compress the adjacent brain parenchyma, resulting in neuronal necrosis and vasogenic edema (30, 31). Platelets, which play a role in clot formation and the inflammatory response, can be elevated in response to inflammatory cytokines and thrombopoietin production, further complicating the disease process (32). Furthermore, iron-deficiency status associated with chronic inflammation may contribute to thrombocytosis through enhanced platelet production (32). Given these processes, the HALP score, calculated using hemoglobin, albumin, lymphocytes, and platelets, is expected to serve as a valuable indicator for predicting clinical outcomes in MUE. However, it is important to acknowledge that the precise pathophysiological pathways linking a lower HALP score to increased MUE mortality remain to be fully elucidated, and further studies are needed to uncover these underlying mechanisms.

Consistent with findings in human medicine, our study demonstrates that a lower HALP score is a significant predictor of short-term mortality in dogs with MUE. While both hemoglobin and albumin levels were individually higher in survivors, the composite HALP score showed a more pronounced association with survival outcomes, suggesting it is a more robust prognostic indicator than any single component. The significance of hemoglobin as a prognostic factor is likely tied to its roles in oxygen delivery and as a marker for chronic inflammation. Anemia, often exacerbated by inflammatory cytokines that suppress erythropoiesis and disrupt iron metabolism (33, 34), can lead to cerebral hypoxia and worsen neurological deficits (35). Thus, higher hemoglobin levels may reflect better systemic health and confer a neuroprotective advantage (36). Similarly, higher albumin levels in survivors point to its importance in prognosis. Chronic inflammation in MUE can increase blood-brain barrier permeability (37, 38), leading to albumin leakage into the CNS, which is associated with poor outcomes. Furthermore, as a negative acute-phase protein, low serum albumin reflects the severity of systemic inflammation. Its inherent neuroprotective properties—including antioxidant and anti-thrombotic effects (39, 40)—also mean that adequate albumin levels may help mitigate neuroinflammation and preserve CNS integrity. In summary, maintaining adequate levels of hemoglobin and albumin appears to be associated with improved clinical outcomes, and the HALP score effectively integrates these key markers of inflammation and systemic health into a single, powerful prognostic tool for dogs with MUE.

The HALP score demonstrated strong predictive performance for mortality. ROC curve analysis revealed good accuracy for predicting three-month mortality (AUC = 0.79) and very good accuracy for one-year mortality (AUC = 0.80), according to established classifications (41). The optimal cut-off values provide direct clinical utility. For three-month mortality, the cut-off of 12.25 yielded high specificity (95.00%), making it effective at identifying dogs with a higher probability of survival. This can help clinicians encourage owner adherence to treatment plans. For one-year mortality, the cut-off of 34.33 provided high sensitivity (95.24%), allowing for the early identification of high-risk patients who may benefit from more intensive management and for whom owners can be given a more guarded prognosis. These prognostic distinctions were validated by Kaplan-Meier analysis. At both the three-month and one-year cut-off points, dogs with HALP scores below the threshold had significantly shorter median survival times (*P* = 0.0007 and *P* = 0.0030, respectively). Collectively, these results identify that the HALP score is a valuable tool for stratifying risk and predicting short-term survival in dogs with MUE.

Given the limited prognostic factors currently available for MUE (8, 42), our findings have significant clinical implications. In stark contrast to the high cost of magnetic resonance imaging and the invasiveness of CSF sampling, the HALP score provides a simple, cost-effective, and non-invasive prognostic tool derived from a routine blood test. The ability to identify dogs with a lower HALP score—and therefore a poorer prognosis—is invaluable. It allows clinicians to provide owners with more accurate prognostic information, which facilitates a shared decision-making process. Faced with a high risk of early mortality, an owner may choose to pursue more aggressive therapies or, conversely, elect for palliative care, thereby avoiding intensive treatments that are unlikely to alter the outcome.

This study has several limitations that should be acknowledged. First, the retrospective nature and small sample size may limit the generalizability of our findings. Larger, prospective multi-center studies are warranted to validate the prognostic utility of the HALP score in a broader population of dogs with MUE. Second, this study did not statistically account for the potential impact of different treatment protocols on survival. While all dogs received immunosuppressive therapy, the specific drug combinations and dosages varied over the 10-year study period. Third, the specific causes of mortality were not fully categorized for all cases. Although most dogs were confirmed to have died or been euthanized due to MUE progression during clinical follow-up, a small subset of cases followed up via telephone interviews may have included deaths from unrelated comorbidities or treatment-related adverse effects. Fourth, while we have proposed potential pathophysiological links, the precise mechanisms connecting a lower HALP score to increased mortality in MUE remain to be fully elucidated. Further research is necessary to explore the complex interplay between the hematological parameters of the HALP score and the neuroinflammatory processes of the disease. Finally, this study did not account for the histopathological subtypes of MUE. Since most cases were presumptively diagnosed without definitive subtyping, we could not assess whether the HALP score's prognostic value differs among MUE subtypes (e.g., granulomatous and necrotizing). Future studies correlating the HALP score with histopathological findings are needed to refine its clinical application.

In conclusion, this study establishes that a lower HALP score is a significant predictor of short-term mortality in dogs with MUE. This positions the HALP score as a practical and accessible prognostic tool that can aid clinicians in risk stratification and guide treatment strategies. Furthermore, because the HALP score integrates key markers of systemic inflammation, nutrition, and immune function, its utility may extend beyond MUE. Future research should explore its potential as a prognostic indicator in other inflammatory and immune-mediated diseases in veterinary medicine.

## Data Availability

The original contributions presented in the study are included in the article/supplementary material, further inquiries can be directed to the corresponding author.
